# Egg Morphology as a Phenotypic Indicator of Chick Sex and Early Growth in Thai Native Chickens

**DOI:** 10.3390/ani16203201

**Published:** 2026-10-12

**Authors:** Wuttigrai Boonkum, Supakorn Authaida, Vibuntita Chankitisakul

**Affiliations:** 1 Department of Animal Science, Faculty of Agriculture, Khon Kaen University, Khon Kaen 40002, Thailand; wuttbo@kku.ac.th (W.B.); supakorn.u@kkumail.com (S.A.); 2 Network Center for Animal Breeding and Omics Research, Khon Kaen University, Khon Kaen 40002, Thailand

**Keywords:** juvenile growth, egg morphology, regression, path analysis, indigenous chicken

## Abstract

Egg morphological characteristics have been associated with hatchability, chick quality, and sex-related variation; however, their relationships with juvenile growth in indigenous chickens remain unclear. This study evaluated associations among egg morphology, hatch weight, chick sex, and early growth in Thai native Pradu Hang Dum chickens. A total of 139 chicks were evaluated for egg length, egg width, egg weight, eggshell surface area, shape index, hatch weight, body weight, and chest circumference. Pearson correlation, multiple linear regression, logistic regression, receiver operating characteristic analysis, and exploratory path analysis were applied. Egg length was positively associated with body weight and chest circumference at 8 weeks, whereas shape index was negatively associated with both traits. Egg weight was strongly correlated with hatch weight (*r* = 0.721, *p* < 0.001). After adjustment for chick sex, egg weight and shape index were not independently associated with body weight at 8 weeks, whereas sex showed a strong association; the model explained 41.0% of the observed variation. For chest circumference, shape index and sex remained significant, and the model explained 18.0% of the variation. Logistic regression showed significant associations between egg morphometric traits and chick sex, with similar within-sample discrimination for shape index alone (AUC = 0.808) and the model combining egg length and width (AUC = 0.814). Exploratory path analysis showed significant paths from egg weight to hatch weight, hatch weight to 8-week body weight, sex to 8-week body weight, and 8-week body weight to chest circumference. Overall, egg morphology provided population-specific phenotypic information associated with chick sex and early development, but its independent value for explaining juvenile growth was limited after accounting for sexual dimorphism.

## Simple Summary

Egg size and shape can be measured easily before incubation and may provide useful information for poultry breeding and hatchery management. This study examined whether these simple egg measurements could help describe differences in chick sex and early growth in Thai native Pradu Hang Dum chickens. The work also explored how egg characteristics may be linked with development from hatching to 8 weeks of age. Understanding these relationships may help identify practical, low-cost indicators that complement conventional performance records. However, egg morphology should be used as supporting information rather than as the sole basis for breeding or management decisions.

## 1. Introduction

Native chicken production plays an important role in sustainable poultry farming, particularly in tropical and developing countries where indigenous genetic resources contribute to food security, rural livelihoods, and biodiversity conservation [1,2]. Among Thai indigenous breeds, Pradu Hang Dum has gained commercial importance because it adapts well to local production conditions and its meat has a distinctive taste and texture that consumers value [3,4,5]. Consequently, breeding programs have emphasized improving productive and reproductive performance while preserving the breed’s unique genetic characteristics [5]. Although breeders have made substantial genetic progress in growth traits, reproductive efficiency and early chick development remain important breeding objectives because they directly influence hatchery efficiency and production profitability [6,7,8].

Egg morphology reflects biological and evolutionary constraints associated with egg formation and species life history [9]. Researchers have investigated traits such as egg length, egg width, egg weight, shell surface area, and egg shape index as non-invasive morphometric features for chick-sex assessment [10,11,12]. Egg characteristics and incubation conditions also affect embryonic development, hatchability, and chick quality [13,14]. Egg weight is consistently recognized as an important determinant of hatch weight because larger eggs provide greater nutrient reserves for embryonic growth [13]. However, the influence of egg morphology on post-hatch growth remains poorly understood, particularly in indigenous chicken populations.

Egg morphology, particularly the egg shape index, has attracted attention as a potential non-invasive phenotypic indicator of chick sex [11,15,16,17,18]. However, avian sex is genetically determined through the ZZ/ZW chromosome system, with males being homogametic (ZZ) and females heterogametic (ZW) [19]. Fertilization occurs in the infundibulum before the subsequent deposition of albumen, shell membranes, and the eggshell [20]; therefore, external egg morphology cannot determine the sex of the developing embryo. Any observed relationship between egg morphology and chick sex should instead be interpreted as a secondary phenotypic association. Such associations may reflect processes related to maternal physiology or egg formation, although the underlying biological mechanism remains unresolved [18]. Previous studies have reported variable relationships between egg morphometric traits and chick sex, suggesting these associations may depend on population and method [11,16,17,18].

Egg morphology may also influence juvenile performance by affecting hatch weight. Hatch weight is widely recognized as an early indicator of subsequent growth because heavier chicks generally possess greater nutrient reserves and improved physiological development [14]. Nevertheless, genetic background and environmental conditions, including incubation and thermal environment, shape juvenile growth [4,7,14]. Consequently, individual egg traits are unlikely to fully explain variation in juvenile growth because post-hatch performance is progressively influenced by numerous biological and environmental factors, including genetic background, maternal effects, nutrition, health status, and management conditions.

Recent advances in statistical methods have improved the investigation of relationships among egg morphology, chick sex, and growth performance. Logistic regression can evaluate associations between continuous morphometric traits and a binary outcome, such as chick sex, using continuous egg morphological predictors [11,15]. For growth traits, multiple linear regression (MLR) estimates the combined effects of multiple egg characteristics while accounting for correlations among predictors [21,22]. Correlation analysis quantifies phenotypic relationships among traits, whereas path analysis and structural equation modeling (SEM) distinguish direct and indirect effects, providing greater insight into complex biological pathways [23,24]. Moreover, these complementary approaches enable evaluation of both predictive performance and the biological relationships linking egg morphology to chick sex and early growth performance.

Despite growing interest in egg morphology, hatchability, chick quality, and post-hatch performance, few studies have simultaneously evaluated relationships among egg characteristics, hatch weight, chick sex, and early growth performance in commercial chickens, and even less evidence exists for native chicken breeds. A better understanding of these relationships may help identify complementary phenotypic indicators that can be evaluated before incubation and integrated with conventional breeding information to support genetic improvement of indigenous chickens.

Therefore, this study aimed to (1) evaluate phenotypic associations among pre-incubation egg morphometric traits, hatch weight, body weight, and chest circumference at 8 weeks in Thai native Pradu Hang Dum chickens; (2) examine associations between egg morphometric traits and chick sex and assess their within-sample discriminatory ability; and (3) explore the relationships among egg weight, hatch weight, body weight at 8 weeks, and chest circumference while accounting for chick sex. We hypothesized that egg morphometric traits would be associated with hatch weight, chick sex, and juvenile body traits, and that egg weight and hatch weight would be sequentially associated with subsequent growth.

## 2. Materials and Methods

All experimental procedures involving animals were reviewed and approved by the Institutional Animal Care and Use Committee (IACUC) of Khon Kaen University, Thailand, and were conducted in accordance with the Guidelines for Animal Care and Use in Research of the National Research Council of Thailand (Approval No. IACUC-KKU-117/68).

### 2.1. Experimental Birds and Management

Sixty healthy 43-week-old Thai native Pradu Hang Dum grandparent breeding hens maintained at the experimental chicken farm of the Network Center for Animal Breeding and Omics Research, Faculty of Agriculture, Khon Kaen University, Thailand, provided eggs for this study. The hens had no history of reproductive disorders and were vaccinated against infectious bronchitis, Newcastle disease, fowl pox, and fowl cholera according to the vaccination program recommended by the Department of Livestock Development, Thailand. Newcastle disease and infectious bronchitis vaccines were administered at 7, 21, and 42 days of age and subsequently at 3-month intervals. Fowl pox vaccine was administered at 35 days of age, whereas fowl cholera vaccine was administered at 42 days of age and subsequently every 3 months.

The birds were reared in an open-sided housing system. During the brooding period (0–4 weeks of age), supplemental heating was provided according to standard farm management practices, and birds received a commercial starter diet containing 19% crude protein and 2900 kcal ME/kg. From 4 to 20 weeks of age, birds were fed a grower diet containing 15% crude protein and 2900 kcal ME/kg. Feed and fresh water were provided ad libitum during these periods. At 20 weeks of age, the hens were transferred to individual battery cages (20 × 45 × 40 cm) in accordance with the animal welfare guidelines of the Department of Livestock Development, Thailand. During the laying period, hens received 110 g/bird/day of a layer diet containing 17% crude protein and 2750 kcal ME/kg, with free access to fresh water. Birds were maintained under natural photoperiod conditions (approximately 12 h light/day) without supplemental lighting.

### 2.2. Egg Collection and Morphometric Measurements

Eggs were collected daily from the 60 breeding hens over a 7-day period. Multiple eggs from the same hen could be included; however, individual maternal identity was not recorded for each egg. Consequently, the statistical analyses could not account for individual-hen contributions or potential clustering of observations within hens. A total of 150 eggs were initially collected. Only clean, intact eggs with a normal shell appearance were considered eligible for incubation. Eggs with shell cracks, abnormal shell texture, deformities, double yolks, or other visible defects were excluded. Three eggs were excluded on this basis, leaving 147 eggs for incubation. Each selected egg was assigned a unique identification number using a non-toxic marker to allow traceability throughout incubation, hatching, and post-hatch evaluation. Before incubation, eggs were individually weighed using a calibrated electronic balance (Model UW620H, Shimadzu Corporation, Kyoto, Japan) with a precision of 0.01 g. Egg length, defined as the maximum distance between the blunt and pointed ends, and egg width, defined as the maximum transverse diameter at the widest portion of the egg, were measured to the nearest 0.01 mm using a digital Vernier caliper (Mitutoyo Corporation, Kawasaki, Kanagawa, Japan). Eggshell surface area (*ESA*, cm^2^) was estimated from egg weight according to Hughes [25] as follows: *ESA* = 3.9782 × Egg weight^0.7056^. Egg shape index (*SI*, %) was calculated as follows: *SI* = (Egg width/Egg length) × 100.

### 2.3. Incubation and Hatching

Following morphometric measurements, eggs were stored at 18–20 °C and 75–80% relative humidity for no longer than 7 days before incubation. A total of 147 eggs were set in a forced-air automatic incubator (Model ROMLUANG-H8000, Romluang Electronics, Chiang Mai, Thailand) maintained at 37.5 ± 0.2 °C and approximately 60% relative humidity during the setter period (days 0–18). Eggs were automatically turned once every hour (24 turns per day) through an angle of approximately 45° to support normal embryonic development.

Egg candling was performed on days 7 and 18 of incubation using a high-intensity LED candling lamp. On day 7, candling identified infertile eggs and early embryonic mortality based on the absence of visible blood vessels or embryonic structures. Of the 147 eggs incubated, 145 were fertile, and two were infertile. Three embryos died during early incubation. On day 18, eggs were candled again to identify late embryonic mortality and confirm embryo viability before transfer to the hatcher; three additional embryos were classified as late embryonic deaths. Eggs identified as infertile or containing dead embryos were removed from subsequent evaluation.

The remaining viable eggs were transferred to a separate hatcher (Model ROMLUANG-H8000, Romluang Electronics, Chiang Mai, Thailand) maintained at 36.8–37.2 °C and 70–75% relative humidity until hatch. A total of 139 chicks successfully hatched and were included in the subsequent analyses. This corresponded to 94.6% of the eggs set and 95.9% of the fertile eggs. Hatchability itself was not analyzed as an outcome; the study focused on relationships among egg morphology, chick sex, and post-hatch traits in successfully hatched chicks.

### 2.4. Chick Growth Measurements and Management

Immediately after hatching, each chick was assigned an identification tag corresponding to its egg identification number to maintain individual traceability. Hatch weight was recorded within 12 h after hatch, before access to feed and water, using a digital balance (Model EPS-202, Zepper, Shanghai, China) with 0.01 g precision.

All chicks were reared under the same management conditions until 8 weeks of age. During the brooding period (0–4 weeks), the target temperature was gradually reduced from approximately 33–35 °C during the first 3 days to 31–33 °C during week 1, 29–31 °C during week 2, 27–29 °C during week 3, and 24–26 °C during week 4, with relative humidity maintained at approximately 60–70%. From 4 to 8 weeks of age, no supplemental heating was provided, and the ambient temperature inside the house averaged 27.12 ± 2.33 °C with a relative humidity of 67.25 ± 4.36%. A photoperiod of 16 h light and 8 h dark was maintained throughout the rearing period. From hatch to 4 weeks of age, chicks were fed a commercial starter diet containing at least 19% crude protein and 3% crude fat, with a maximum crude fiber content of 5%. From 4 to 8 weeks of age, chicks received a commercial grower diet containing at least 15% crude protein and 2% crude fat, with a maximum crude fiber content of 6%. Feed and fresh drinking water were provided ad libitum throughout the rearing period.

At 8 weeks of age, individual body weight was measured using a mechanical spring scale (Twin Dolls Brand, Bangkok, Thailand) with 10 g precision. The 8-week measurement was selected because this age represents the routine juvenile evaluation stage used in the Thai native chicken breeding program at the experimental farm. Chest circumference was measured to the nearest 0.1 cm using a flexible measuring tape placed immediately posterior to the wings and around the widest region of the thorax while the bird was standing in a normal upright position.

### 2.5. Sex Determination

**Figure 1:**
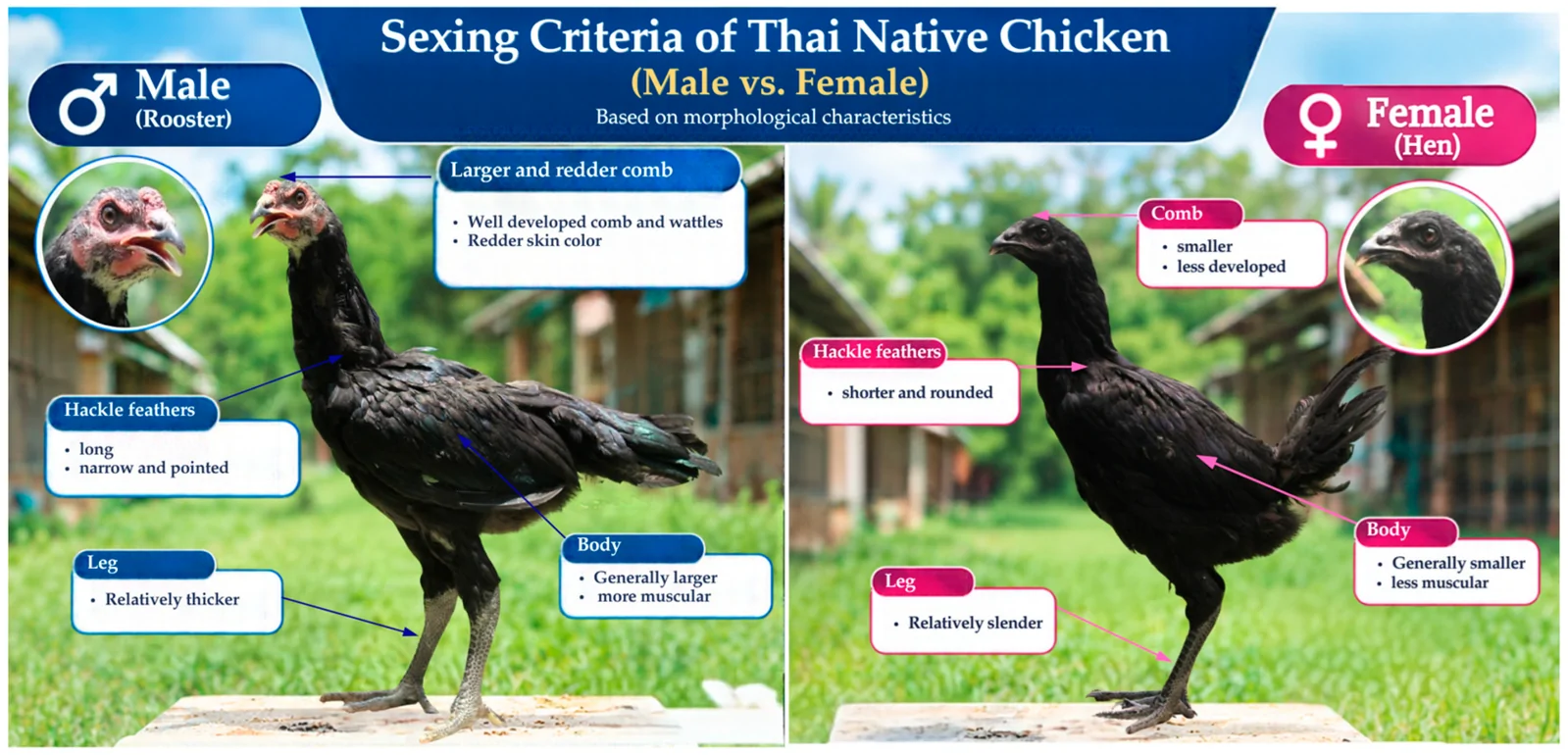
Representative secondary sexual characteristics used to distinguish male and female Thai native Pradu Hang Dum chickens at 8 weeks of age. Sex classification was based on differences in comb and wattle development, plumage characteristics, leg conformation, and overall body development.

Chick sex was determined at 8 weeks of age by an experienced poultry technician based on clearly expressed secondary sexual characteristics, including comb and wattle development, plumage characteristics, and crowing behavior in males, as shown in Figure 1. Sex was recorded as male or female and coded as a binary variable (male = 1, female = 0) for statistical analyses.

### 2.6. Statistical Analysis

All statistical analyses were performed using SAS OnDemand for Academics (SAS 9.4; SAS Institute Inc., Cary, NC, USA). Statistical significance was declared at *p* < 0.05. Descriptive statistics, including the mean, standard deviation (SD), minimum, maximum, and coefficient of variation (CV), were calculated for egg morphological and growth traits.

Pearson correlation coefficients were calculated to evaluate pairwise relationships among egg length, egg width, shape index, egg weight, eggshell surface area, hatch weight, body weight at 8 weeks, and chest circumference at 8 weeks. Pairwise complete observations were used for correlations involving hatch weight. The correlation matrix was visualized as a heatmap.

Multiple linear regression analyses were performed to evaluate the independent associations of pre-incubation egg morphology with body weight and chest circumference at 8 weeks while accounting for chick sex. Egg weight and shape index were retained to represent egg size and egg shape, respectively. To reduce mathematical redundancy among predictors, eggshell surface area was excluded because it was derived directly from egg weight, while egg length and egg width were not included simultaneously with shape index because shape index was calculated from these two dimensions. Because body weight and chest circumference at this age may be influenced by sexual dimorphism, chick sex was included as a fixed covariate in both models. The regression model was expressed as:

*Y* = *β*_0_ + *β*_1_(Egg weight) + *β*_2_(Shape index) + *β*_3_(Sex) + *ε*

where *Y* represents either body weight or chest circumference at 8 weeks of age, *β*_0_ is the intercept, *β*_1_ − *β*_3_ are the regression coefficients, and *ε* is the random error term. Chick sex was coded as male = 1 and female = 0.

Logistic regression analysis was used to evaluate associations between egg morphometric traits and chick sex. To avoid mathematical redundancy among predictors, two alternative models were evaluated. The first model included shape index as a single derived measure of egg shape, whereas the second included egg length and egg width as the original morphometric measurements. Shape index was not entered simultaneously with egg length and egg width because it is calculated directly from these measurements. Chick sex was treated as a binary response variable, with males coded as 1 and females as 0. Regression coefficients (*β*), standard errors (SEs), odds ratios (ORs), 95% confidence intervals (CIs) for the OR, and *p*-values are reported. Overall model performance was evaluated using the likelihood ratio chi-square test, McFadden’s pseudo-*R*^2^, and receiver operating characteristic (ROC) curve analysis. The area under the ROC curve (AUC) was used to assess within-sample discrimination between male and female chicks.

An exploratory path analysis was performed to examine the relationships among egg weight, hatch weight, body weight at 8 weeks, and chest circumference. Chick sex was included as an exogenous covariate to account for sexual dimorphism in the post-hatch traits. The hypothesized pathway was specified as egg weight → hatch weight → body weight at 8 weeks → chest circumference, with additional paths from sex to body weight and chest circumference. Standardized path coefficients were estimated using the maximum likelihood method. Model fit was evaluated using the chi-square test (*χ*^2^), Comparative Fit Index (CFI), Root Mean Square Error of Approximation (RMSEA), and Standardized Root Mean Square Residual (SRMR).

## 3. Results

### 3.1. Descriptive Statistics

Table 1 presents descriptive statistics for egg morphology and early growth traits. Egg morphological traits showed relatively low phenotypic variation, whereas post-hatch body weight varied more. The final study population comprised 75 males (54.0%) and 64 females (46.0%). Among the egg traits, egg weight showed the greatest variability, while body weight at 8 weeks exhibited the highest coefficient of variation among all measured traits.

### 3.2. Pearson Correlation Analysis

**Figure 2:**
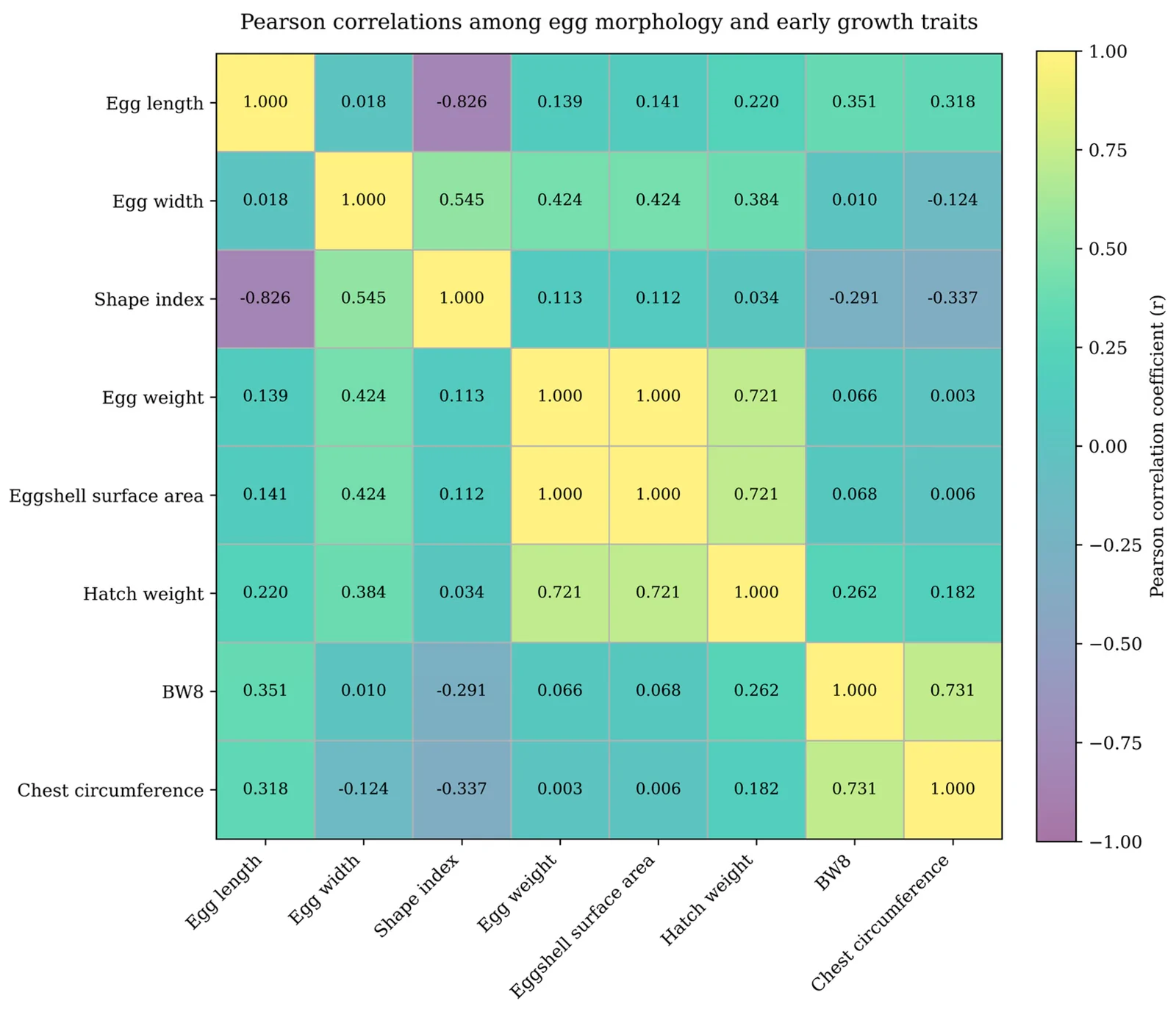
Pearson correlation coefficients among egg morphological traits, hatch weight, and early growth traits in Thai native Pradu Hang Dum chickens.

Pearson correlation analysis revealed several notable relationships among egg morphology and post-hatch traits (Figure 2). Egg length was positively associated with body weight and chest circumference at 8 weeks, whereas shape index was negatively associated with both traits. Egg weight was strongly positively correlated with hatch weight (*r* = 0.721, *p* < 0.001). Hatch weight showed weaker but significant positive correlations with body weight and chest circumference at 8 weeks. As expected, egg weight and the derived eggshell surface area were nearly perfectly correlated. Among post-hatch traits, body weight and chest circumference at 8 weeks showed a strong positive correlation (*r* = 0.731, *p* < 0.001).

### 3.3. Multiple Linear and Logistic Regression Analyses

**Figure 3:**
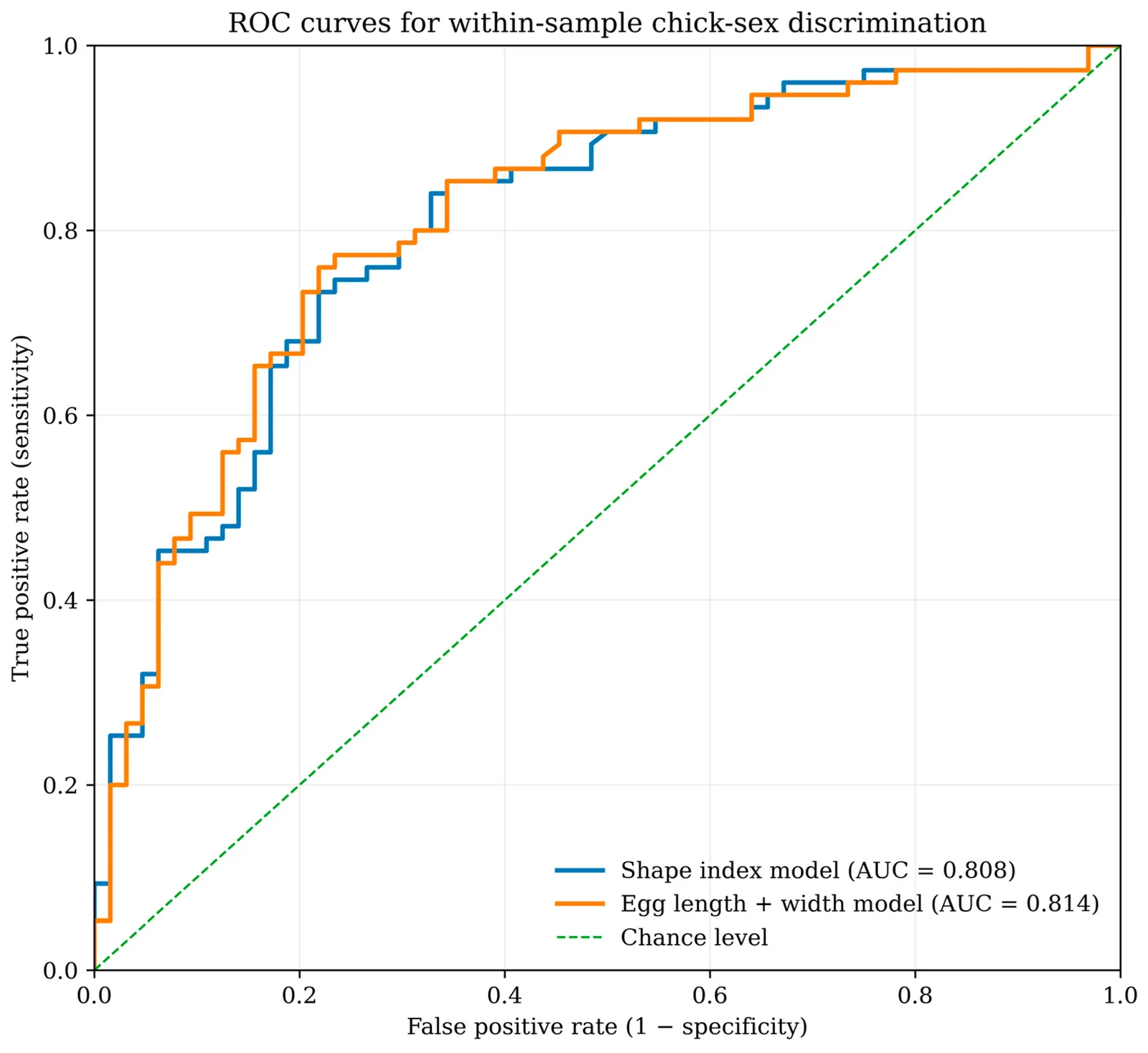
Receiver operating characteristic (ROC) curves comparing the within-sample discrimination of chick sex using shape index alone and a model combining egg length and egg width in Thai native Pradu Hang Dum chickens. The corresponding areas under the curve (AUCs) were 0.808 and 0.814, respectively.

After adjustment for chick sex, the multiple linear regression model for body weight at 8 weeks was significant and explained 41.0% of the observed variation (Table 2). Sex was strongly associated with body weight, whereas egg weight and shape index showed no independent associations with this trait. The model for chest circumference was also significant but explained a smaller proportion of the variation (18.0%; Table 3). Sex remained significantly associated with chest circumference, and shape index showed a modest negative association, whereas egg weight was not significant. Logistic regression analyses showed significant associations between egg morphometric traits and chick sex (Table 4). Shape index alone yielded an AUC of 0.808, whereas the model combining egg length and egg width yielded an AUC of 0.814. Figure 3 presents the corresponding receiver operating characteristic (ROC) curves.

### 3.4. Path Analysis

Exploratory path analysis was conducted using 136 observations (Table 5). Egg weight was positively associated with hatch weight (standardized *β* = 0.721, *p* < 0.001). Hatch weight and chick sex were both positively associated with body weight at 8 weeks (*β* = 0.172, *p* = 0.010 and β = 0.613, *p* < 0.001, respectively). Body weight at 8 weeks was positively associated with chest circumference (*β* = 0.805, *p* < 0.001), whereas the direct path from sex to chest circumference was not significant (*p* = 0.104). Model fit statistics were *χ*^2^ = 9.059 (df = 4, *p* = 0.060), CFI = 0.982, RMSEA = 0.097, and SRMR = 0.067.

## 4. Discussion

This study evaluated relationships among egg morphology, hatch weight, chick sex, and early growth in Thai native Pradu Hang Dum chickens using correlation, regression, logistic regression, and exploratory path analyses. The results showed that several egg morphometric traits were associated with post-hatch characteristics at the phenotypic level; however, their independent associations with juvenile growth decreased after accounting for chick sex. Egg weight was strongly associated with hatch weight, whereas chick sex showed a pronounced association with body weight at 8 weeks. Egg morphometric traits were also associated with chick sex, although the two evaluated logistic models showed similar within-sample discriminatory ability. The exploratory path model further indicated sequential relationships among egg weight, hatch weight, and subsequent growth.

Egg morphological traits showed relatively limited phenotypic variation compared with body weight at 8 weeks. The comparatively large variation in body weight at 8 weeks likely reflected, at least in part, sexual dimorphism within the mixed-sex population, as supported by the strong association between chick sex and body weight in the adjusted regression model. Additional variation may also have arisen from individual genetic and post-hatch environmental differences [26,27,28,29]. Correlation analysis nevertheless identified associations between egg morphology and later body traits. Egg length was positively associated with body weight and chest circumference, whereas shape index was negatively associated with these traits. Egg weight was strongly associated with hatch weight, consistent with previous studies showing that heavier eggs generally produce heavier hatchlings because they provide greater nutrient reserves during embryonic development [13,30,31]. Hatch weight, however, showed only weak associations with body weight and chest circumference at 8 weeks, indicating that the relationship observed at hatch became less pronounced by 8 weeks in the present population.

Multivariable analyses also showed that chick sex substantially influenced the associations between egg morphology and juvenile growth. Sexual dimorphism in juvenile growth has been reported in chickens, including Thai native Pradu Hang Dum chickens, with males generally exhibiting greater body weight than females as growth progresses [28,29]. After adjustment for sex, neither egg weight nor shape index was independently associated with body weight at 8 weeks, whereas sex showed a strong association with body weight. This finding suggests that part of the unadjusted relationship between egg morphology and body weight may have reflected sex-related differences in juvenile growth rather than an independent association between egg morphology and subsequent body weight. In contrast, shape index retained a modest negative association with chest circumference after adjustment for sex, suggesting that egg shape may contain phenotypic information related to subsequent body conformation beyond what sex explains. Nevertheless, the relatively small magnitude of this association, together with the absence of an independent association with body weight, indicates that egg morphology alone has limited value for explaining individual variation in juvenile growth and should not be considered an independent selection criterion for growth performance.

The exploratory path analysis provided further information on the relationships among egg weight, hatch weight, sex, and subsequent growth. Egg weight showed a strong positive association with hatch weight, consistent with the established relationship between egg size and chick weight at hatch [13,30,31]. Hatch weight was subsequently associated with body weight at 8 weeks, although this pathway was considerably weaker than the association between sex and body weight. Body weight at 8 weeks, in turn, was strongly associated with chest circumference. The direct path from sex to chest circumference was not significant after including body weight in the model. These findings are consistent with a developmental sequence in which egg weight is related primarily to hatch weight, whereas later juvenile growth is increasingly associated with other factors, including sex.

Model-fit indices were not uniformly optimal. The chi-square test was non-significant, CFI and SRMR indicated satisfactory fit, and RMSEA remained above the commonly recommended threshold. Accordingly, the path model should be regarded as exploratory rather than as evidence of definitive causal relationships. Additional maternal, genetic, nutritional, incubation, and post-hatch environmental factors that were not represented in the present model may also contribute to variation in juvenile growth.

Egg morphometric traits were significantly associated with chick sex. Shape index alone showed an AUC of 0.808, whereas the model based on egg length and width yielded an AUC of 0.814. The small difference indicates that the two models had similar within-sample discriminatory ability. Importantly, this difference in AUC should not be interpreted as evidence of a meaningful improvement in practical discriminatory performance, particularly because neither model was externally validated. Previous studies have likewise reported associations between external egg morphology and chick sex, although the magnitude and consistency of these relationships vary among breeds and populations [11,12,15,18].

These findings suggest that sex-associated variation in external egg morphology may involve multiple morphometric characteristics rather than a single egg trait. Importantly, however, these associations should not be interpreted as causal. In chickens, genetic sex is established at fertilization through the ZZ/ZW sex-chromosome system, with males being homogametic (ZZ) and females heterogametic (ZW) [19]. Fertilization occurs in the infundibulum before the egg acquires albumen, shell membranes, and the calcified shell as it passes through the oviduct [20]. Consequently, external egg morphology cannot determine the sex of the developing embryo. Instead, morphometric differences between eggs producing male and female chicks should be regarded as secondary phenotypic associations that may reflect processes related to maternal or oviductal physiology during egg formation, although the underlying biological mechanisms remain unresolved. Recent evidence further indicates that relationships between egg morphometric traits and chick sex may vary among chicken populations, supporting a cautious, population-specific interpretation of these associations [18].

Several limitations of the present study warrant acknowledgment. First, the analytical dataset did not retain individual maternal identity; therefore, the statistical models could not explicitly account for potential maternal effects and non-independence among eggs from the same hen. Accordingly, potential within-hen dependence should be considered when interpreting the estimated associations and their statistical significance. Second, the study was conducted in a single Thai native Pradu Hang Dum population with a relatively limited sample size, which may restrict the generalizability of the observed associations. Third, growth was evaluated at hatch and at 8 weeks of age rather than through repeated measurements across the entire juvenile growth period. In addition, the chick-sex classification models were evaluated within the same study population and were not externally validated. Future studies should therefore retain dam identification, include larger and genetically diverse populations, collect repeated growth measurements, and validate the observed associations in independent datasets.

Despite these limitations, external egg measurements remain attractive because they are inexpensive and non-invasive. The present results indicate that such measurements may provide complementary phenotypic information on hatch weight, body conformation, and sex-associated variation, but their utility for growth selection or pre-hatch sex classification should not be assumed without further validation.

## 5. Conclusions

This study demonstrated that egg morphological traits were associated with hatch weight, chick sex, and juvenile body traits in Thai native Pradu Hang Dum chickens. However, after accounting for chick sex, egg weight and shape index were not independently associated with body weight at 8 weeks, whereas shape index retained only a modest association with chest circumference. Egg morphometric traits also discriminated chick sex within samples, with similar AUC values for shape index alone and the model combining egg length and width. Exploratory path analysis identified sequential associations among egg weight, hatch weight, body weight at 8 weeks, and chest circumference, while chick sex showed a strong association with juvenile body weight. Because the path model showed mixed fit indices and the chick-sex models were not externally validated, these findings should be interpreted cautiously. Overall, egg morphology may provide complementary phenotypic information but should not be considered a standalone predictor or independent selection criterion. Validation in larger and genetically diverse populations is warranted.

## Figures and Tables

**Table 1:** Descriptive statistics of egg morphology and early growth traits in Thai native Pradu Hang Dum chickens (*n* = 139 for all traits).

Trait	Mean ± SD	Min	Max	CV (%)
Egg length (mm)	53.26 ± 2.67	48.10	62.40	5.01
Egg width (mm)	40.98 ± 1.35	37.20	44.00	3.30
Shape index (%)	77.14 ± 4.49	63.40	89.70	5.82
Egg weight (g)	50.04 ± 3.63	41.00	61.00	7.25
Eggshell surface area (cm^2^)	64.43 ± 3.09	56.50	73.50	4.79
Hatch weight (g)	33.90 ± 2.58	27.00	41.00	7.62
Body weight at 8 weeks (g)	840.32 ± 130.83	520.00	1120.00	15.57
Chest circumference at 8 weeks (cm)	20.02 ± 1.43	15.50	23.50	7.15

**Table 2:** Multiple linear regression analysis of body weight at 8 weeks of age after adjustment for chick sex.

Predictor	Regression Coefficient (*β*)	SE	95%CI	*p*-Value
Intercept	558.98	202.94	157.62 to 960.34	0.007
Egg weight (g)	2.47	2.40	−2.29 to 7.22	0.307
Shape index (%)	0.86	2.25	−3.59 to 5.30	0.704
Sex (male vs. female)	170.28	20.04	130.64 to 209.92	<0.001
Model statistics				
*R* ^2^				0.410
Adjusted *R*^2^				0.397
*p*-value				<0.001

**Table 3:** Multiple linear regression analysis of chest circumference at 8 weeks of age after adjustment for chick sex.

Predictor	Regression Coefficient (*β*)	SE	95%CI	*p*-Value
Intercept	23.72	2.62	18.54 to 28.90	<0.001
Egg weight (g)	0.011	0.031	−0.051 to 0.072	0.728
Shape index (%)	−0.061	0.029	−0.118 to −0.003	0.038
Sex (male vs. female)	0.848	0.259	0.336 to 1.359	0.001
Model statistics				
*R* ^2^				0.180
Adjusted *R*^2^				0.162
*p*-value				<0.001

**Table 4:** Logistic regression analysis of the association between egg morphometric traits and chick sex.

Model	Predictor	*β*	SE	Odds Ratio	95% CI	*p*-Value
Shape index model	Shape index (%)	−0.325	0.062	0.723	0.640 to 0.817	<0.001
Egg dimensions model	Egg length (mm)	0.499	0.100	1.647	1.353 to 2.004	<0.001
	Egg width (mm)	−0.518	0.172	0.596	0.426 to 0.834	0.003
Model statistics						
Shape index model						
Likelihood ratio χ^2^					42.13	
*p*-value					<0.001	
McFadden pseudo-*R*^2^					0.220	
AUC					0.808	
Egg dimensions model						
Likelihood ratio χ^2^					42.60	
*p*-value					<0.001	
McFadden pseudo-*R*^2^					0.222	
AUC					0.814	

**Table 5:** Standardized direct path coefficients and model goodness-of-fit indices from the exploratory path model describing relationships among egg weight, hatch weight, sex, and early growth traits in Thai native Pradu Hang Dum chickens.

Path	Standardized Coefficient	SE	CR	*p*-Value
Egg weight → Hatch weight	0.721	0.060	12.038	<0.001
Hatch weight → BW8	0.172	0.066	2.612	0.010
Sex → BW8	0.613	0.066	9.324	<0.001
BW8 → Chest circumference	0.805	0.077	10.481	<0.001
Sex → Chest circumference	−0.126	0.077	−1.637	0.104
Model fit				
*χ* ^2^	9.059			
df	4			
*χ*^2^ test *p*-value	0.060			
CFI	0.982			
RMSEA	0.097			
SRMR	0.067			

BW8, body weight at 8 weeks of age; SE, standard error; CR, critical ratio; CFI, comparative fit index; RMSEA, root mean square error of approximation; SRMR, standardized root mean square residual. Sex was coded as male = 1 and female = 0.

## Data Availability

The original contributions presented in this study are included in this article; further inquiries can be directed to the corresponding author.
